# Activation of the anterior cingulate cortex ameliorates anxiety in a preclinical model of fetal alcohol spectrum disorders

**DOI:** 10.1038/s41398-022-01789-1

**Published:** 2022-01-20

**Authors:** Hye M. Hwang, Kazue Hashimoto-Torii

**Affiliations:** 1 Center for Neuroscience Research, The Children’s Research Institute, Children’s National Hospital, Washington, DC USA; 2 The Institute for Biomedical Sciences, School of Medicine and Health Sciences, The George Washington University, Washington, DC USA; 3 Departments of Pediatrics, and Pharmacology & Physiology, School of Medicine and Health Sciences, The George Washington University, Washington, DC USA

**Keywords:** Neuroscience, Psychiatric disorders

## Abstract

People with fetal alcohol spectrum disorders (FASD) are suffered from a wide range of interlinked cognitive and psychological problems. However, few therapeutic options are available for those patients due to limited dissection of its underlying etiology. Here we found that prenatal alcohol exposure (PAE) increases anxiety in mice due to a dysregulated functional connectivity between the anterior cingulate cortex (ACC) and basolateral amygdala (BLA). We also show that chemogenetic activation of excitatory neurons in the ACC reduced this anxiety behavior in the PAE mice. Interestingly, although the level of plasma corticosterone correlated with the increase in anxiety in the PAE, this level was not altered by chemogenetic activation of the ACC, suggesting that the functional connectivity between the ACC and the BLA does not alter the activity of the hypothalamic–pituitary–adrenal axis. Altogether, this study demonstrated that reduced excitation in the ACC is a cause of anxiety in the PAE mice, providing critical insights into the ACC–BLA neural circuit as a potential target for treating anxiety in FASD patients.

## Introduction

Prenatal alcohol exposure (PAE) results in a broad range of neurobehavioral issues, including cognitive impairment and attention deficits, in patients with fetal alcohol spectrum disorders (FASD) [1–3]. One prominent lifelong comorbidity in these patients is anxiety [4–8]. Both preclinical and clinical studies suggested that disrupted hypothalamic–pituitary–adrenal (HPA) axis might be one of the reasons for increased anxiety in FASD [9–14], and this disruption was detected as early as 5 months after birth [11]. However, changes in brain circuitry or molecular pathways other than the HPA axis, have not yet been elucidated in FASD.

One of the brain regions that is highly associated with anxiety is the anterior cingulate cortex (ACC). Malfunctioning of the ACC was associated with hyperactivity of the amygdala, a hub brain region for fear processing, in patients with anxiety disorders [15, 16]. Conversely, stimulation of the ACC led to a decreased neuronal activity in the amygdala [17]. In innate fear response in mice, the activity of the basolateral amygdala (BLA) was modulated by glutamatergic projecting neurons from the ACC [18]. The association of the ACC–BLA network with innate behavior suggests a possibility that the effects of PAE on this circuitry underlie the anxiety in the FASD. In fact, gross structural changes in the ACC, such as reduction of the surface area and volumes of both grey and white matter, were observed in the FASD patients [19, 20]. However, the association of these changes with anxiety behavior, if any, is still unknown.

Here, we demonstrate that mice with acute alcohol exposure during late corticogenesis show anxiety-like behavior, as seen in FASD patients, due to reduced functional connectivity between ACC and BLA resulting from an imbalance of inhibitory and excitatory local network activity in the ACC.

## Materials and methods

### Animals

Pregnant CD-1 mice were purchased from Charles River and housed under a standard 12 h light/dark cycle (6 AM-6 PM) in a temperature and humidity-controlled vivarium. Pregnant CD-1 mice were administered with 4.0 g/kg of 25% ethanol in phosphate-buffered saline (PBS) (PAE) or an equal volume of PBS for control mice via intraperitoneal injection (i.p.) at E16 and E17. Pups were weaned at P21 and group-housed until the day of the behavioral experiment. For naïve control and PAE mice, PBS or 25% ethanol was injected via i.p. to pregnant CD-1 mice as described earlier and sacrificed at P30 or P90. Food and water were available ad libitum throughout the experiment. For all of the experiments, the animals were randomly assigned to each group without any pre-determined criteria. All animal protocols were approved by Institutional Animal Care and Use Committee at Children’s National Hospital and complied with ethical regulations.

### Self-grooming test

Each mouse was put in a clear Plexiglas chamber (8 cm × 20 cm × 20 cm) and underwent video recording for 300 s. The total time of self-grooming, the number of self-grooming events (frequency), and the duration of self-grooming per event (duration) were manually analyzed from the recorded videos.

### Elevated plus maze (EPM) test

The maze is a grey plus-shaped apparatus with two open arms and two closed arms linked by a central platform. Mice were individually put in the center of the maze facing an open arm and allowed to explore the maze for 300 s. A video was recorded during the experiment and analyzed with MoutBeat ImageJ Plugin as per the user guide [21].

### Tissue processing

All mice were anesthetized with isoflurane and fixed by intracardiac perfusion of 4% paraformaldehyde in PBS at pH 7.4. Brains were quickly removed, postfixed overnight in the same fixative solution, and then sequentially incubated in 10% sucrose and 30% sucrose solutions (in PBS) for 24 h each. After incubation with 30% sucrose solution, brains were embedded in the OCT compound (VWR, Radnor, PA, cat# 4583), cryosectioned at 50 µm, and stored at −20 °C in a cryoprotective solution until further analysis.

### Immunohistochemistry

Brain sections measuring 50 µm were washed with 1× PBS, and antigen retrieval was performed according to the manufacturer’s protocol (Invitrogen, Waltham, MA, cat# 00-4955-58). Endogenous peroxidase activity was blocked by incubating in H_2_O_2_/methanol (1:4) for 30 min at −20 °C. The sections were then incubated in 2% bovine serum albumin prepared in 1× PBS for blocking nonspecific interactions. These brain sections were then incubated overnight at 4 °C on a shaker with primary antibodies against c-Fos E-8 (1:100; Santa Cruz, Dallas, TX, cat# sc-166940), VGLUT2 (1:100; Sigma-Aldrich, St. Louis, MO, cat# MAB5504), PV (1:100; Sigma-Aldrich, cat# P3088), somatostatin (SST) (1:50; EMD Millipore, Burlington, MA, cat# MAB354), Calretinin (1:500; Abcam, Cambridge, MA, cat# AB244299), vasoactive intestinal peptide (VIP) (1:100; Thermo Fisher, Waltham, MA, cat# PA5-78224), CamKII(pan) (1:100, Cell Signaling, Danvers, MA, cat# 3362), and/or mCherry (1:200; Abcam, cat# ab167453). After washing, sections were incubated in HRP-conjugated secondary antibodies at 1:300 dilution at room temperature for 2 h on a shaker (HRP-conjugated anti-mouse IgG; Jackson Immuno, West Grove, PA, cat# 111-035-146, HRP-conjugated anti-rabbit IgG; Jackson Immuno cat# 111-035-144, HRP-conjugated anti-rat IgG; Jackson Immuno, cat#112-035-003), followed by a 1-h incubation with TSA Plus Cyanine-2 (Akoya, Marlborough, MA, cat# NEL741001KT) or Cyanine-3 (Akoya, cat# NEL753001KT) diluted at 1:300. For c-Fos E-8 primary antibody, anti-mouse IgG Alexa Fluor Plus 647 (Invitrogen, cat# A32787) secondary antibody diluted at 1:500 was used for 1-h incubation at room temperature on a shaker. All brain sections were counter-stained with DAPI (1:10,000) and mounted onto glass slides with CC/Mount (Sigma-Aldrich, cat# C9368) mounting medium. Images were acquired with either Olympus (Center Valley, PA) BX63 or FV1000 inverted confocal microscopes. Cell counter plugin for Image J was used for quantification. Adjustments of brightness and contrast were applied equally across the images in all experimental groups.

### Plasma collection

Mice were anesthetized with isoflurane, and whole blood was collected by cardiac puncture using a 23–25 G needle and transferred to an EDTA-coated tube (BD, Franklin Lakes, NJ, cat#365974). Tubes were inverted a few times and kept on ice until centrifugation. Tubes were centrifuged at 2000*g* for 10 min at 4 °C to separate plasma. Separated plasma was transferred to clean 1.5 ml centrifuge tubes, snap-frozen in liquid nitrogen, and stored in a −80° freezer until further analysis.

### The enzyme-linked immunosorbent assay (ELISA)

Plasma corticosterone was measured using the Corticosterone ELISA kit (Crystal Chem, Elk Grove Village, IL, cat #80556, lot# 9218) as per the manufacturer’s protocol.

### Viral injection

Injections were done at P1 with AAV5-CaMKIIa-hM3D(Gq)-mCherry (Addgene, Watertown, MA, cat# 50476) at 10^12^–10^13^ particles/ml as per the procedure described by Teissier et al. [22]. Briefly, the anesthetized pup was kept in a prone position with the skull flat. The skin was gently opened as a single incision with a razor blade, and the mPFC was targeted using the inferior cerebral vein and the superior sagittal sinus as a reference. Bilateral injections (200 nl each) were performed with a nanoinjector (Drummond Scientific Company, Broomall, PA, cat#3-000-207). The glass capillary was held in place for 1 min following injection to reduce the backflow of the virus. Then the opened skin was sealed with tissue glue (Fisher Scientific, Waltham, MA, cat#NC9259532), and the pup was gently warmed up. All pups from the same litter were injected to avoid competition for feeding and returned together to their home cage at the end of the procedure.

### Administration of clozapine N-oxide (CNO)

CNO (Sigma-Aldrich, cat#SML-2304) at 3 mg/kg prepared in PBS or vehicle (PBS solution) was injected intraperitoneally 30 min prior to the behavioral experiment.

### Statistics

Statistical analyses were performed using GraphPad Prism (Version 7.01). The sample size for each experiment was determined based on previous experience in similar experiments. All data were assessed for normality with D’Agostino & Pearson normality (for experiments with *n* ≧ 8 samples) or Shapiro–Wilk normality tests (*n* < 8). The statistical test was chosen accordingly. Using the GraphPad prism, we tested the equal variances in our data. Two-way ANOVA followed by simple main effects test was used when there was a statistically significant interaction between two factors, and Tukey’s post hoc test was used otherwise. In addition, unpaired two-tailed *t*-tests were used when comparing two groups, as indicated in the figure legends. For all results, the significance threshold was placed at *p* < 0.05. Data collection or analyses was not performed blind to the conditions of the experiments. However, equal parameters and processes were applied to all groups, and data were analyzed in an automated manner whenever possible. In DREADD experiments, animals with mCherry expression outside of ACC were excluded from the analyses. All behavior experiments and immunostaining were repeated at least twice with the number of animals indicated in the figure legends that were pooled from at least two different litters. For ELISA experiments, all samples and standards were run twice to make sure reproducibility.

## Results

### Acute PAE during late corticogenesis leads to anxiety phenotype in mice

**Fig. 1:**
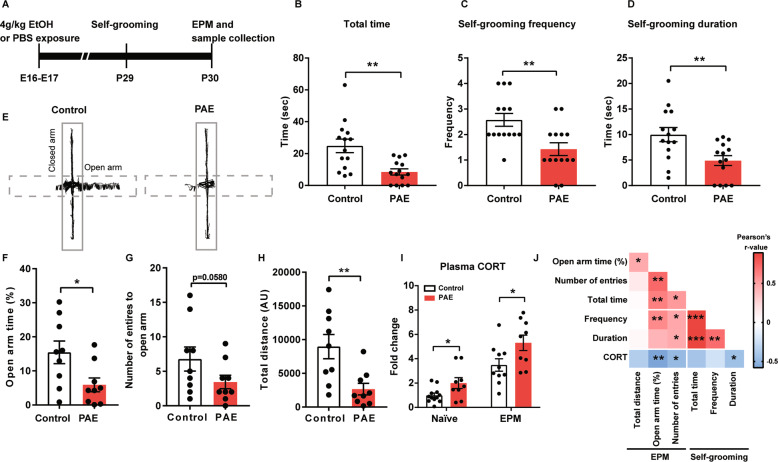
PAE mice show increased anxiety and corticosterone in the plasma. **A** Experimental timeline. **B**–**D** PAE mice show a significant decrease in total time (**B**, *p* = 0.0015), frequency (**C**, *p* = 0.0034), and duration (D, *p* = 0.0057) of self-grooming compared to control mice. *n* = 14 per group. **E** Representative images of line tracks generated by MouBeat ImageJ plug-in from a recording of a mouse in an EPM test. Dashed and solid boxes outline open and closed arms, respectively. **F**–**H** Time in open arm (**F**, *p* = 0.0259), the number of entries to open arm (**G**, *p* = 0.0580), and total travel distance (**H**, *p* = 0.0059) are significantly reduced in the PAE mice. *n* = 9 per group. **I** Fold change of the plasma corticosterone (CORT) concentration was compared to the naïve control group (control mice that were not placed in any behavior tests). In both naïve conditions and 60 min post EPM test, PAE mice show a significant increase in plasma CORT level compared to the control mice (*p* = 0.0332 and *p* = 0.0192, respectively). Naïve: Control *n* = 12, PAE *n* = 9, EPM: Control *n* = 10, PAE *n* = 9. **J** A correlation matrix is presented to show associations between variables of behavior tests (self-grooming and EPM) and concentrations of plasma CORT. *,**,*** = *p* < 0.05, 0.01, 0.001 by one-tailed Pearson’s correlation coefficient. **B**–**D**, **F**–**I** Graphs represent mean ± SEM, and each dot represents an individual animal. Unpaired two-tailed Student’s *t*-test was used for statistical analyses. *,**p < 0.05, 0.01.

Generation of the FASD mouse model was performed according to a previously published protocol [23, 24], where pregnant CD-1 mice received an intraperitoneal injection (i.p.) of 25% ethanol (in PBS) solution at 4.0 g/kg bodyweight at embryonic day (E) 16 and 17. For the control group, an equal volume of PBS was injected instead on the same days. To measure anxiety, we used self-grooming and the elevated plus maze (EPM) behavioral tests. Self-grooming and the EPM tests were conducted with the offspring at postnatal day (P) 29 and 30, respectively, as outlined in Fig. 1A, and 300-second video recordings were performed during behavioral tests to analyze mouse behavior.

Self-grooming is an innate behavior of rodents and shown to be reduced by anxiety [25]. Manually analyzed 300-s video recordings revealed that the total time spent on self-grooming was significantly lower in the PAE mice (Fig. 1B). Similar results were reported in the mouse model of a chronic PAE [26]. We also quantified the total number of self-grooming events (frequency) and average time spent per grooming event (duration) during the recording and found that these measurements were also significantly decreased by PAE (Fig. 1C, D).

The EPM test is widely used for measuring anxiety-like behavior in rodents. The 300-s video recordings were analyzed with Mouse Behavioral Analysis Toolbox (MouBeAT) in ImageJ as outlined by a previous paper [21]. The analysis showed that the PAE mice spent a substantially shorter time in the open arm with fewer entries to the arm (Fig. 1E–G), indicating elevated anxiety. These results are consistent with previous animal studies that indicate heightened anxiety and fear due to PAE [27–29]. The PAE mice also traveled a significantly shorter distance in the maze (Fig. 1H), indicating reduced exploratory behavior. This mouse model does not have any locomotive problems [23], and therefore the shorter distance traveled by PAE mice in the EPM maze is likely due to elevated anxiety rather than problems with locomotion.

In response to stress, the HPA axis is activated, and stress hormones, such as corticosterone (CORT), are released. Therefore plasma samples were collected from naïve control and PAE mice that were not placed in any behavior tests and 60 min post EPM test when CORT release peaks after stressful behavioral tests [30]. The PAE mice showed a significant increase in their CORT level compared to control after the EPM test (Fig. 1I), suggesting an increased HPA axis response. Similarly, a significant increase of CORT in the PAE mice was also observed in the naïve group (Fig. 1I), indicating a higher baseline of CORT level in the PAE mice. Those results are consistent with results obtained from a rat PAE model and human FASD patients [13, 28, 31]. Correlation analyses were also performed to discover associations between anxiety-like behaviors and CORT concentration in plasma. We found that plasma CORT concentration inversely correlated with time spent in the open arm, the number of entries to the open arm in the EPM, and duration of self-grooming (Fig. 1J), suggesting that level of CORT in plasma is associated with anxiety-like behavior. As anticipated from previous findings [32], significant correlations were also observed between measures collected from two behavior tests, the EPM and self-grooming tests (Fig. 1J).

Sexual dimorphism has been implied in anxiety behavior and CORT release in FASD [31, 33, 34]. Therefore, independent statistical analysis was performed to determine if any sex-specific differences exist between males and females in the PAE mice. We found no differences in self-grooming behavior (Supplementary Fig. 1A), EPM test (Supplementary Fig. 1B), or fold change of CORT concentration (Supplementary Fig. 1C) between males and females in either the PAE or control mice. Therefore, we concluded that there is no sexual dimorphism, as it relates to anxiety-like behavior and plasma corticosterone concentrations, in our FASD model.

### Neuronal activity is altered in ACC input to BLA neurons in the PAE mice

**Fig. 2:**
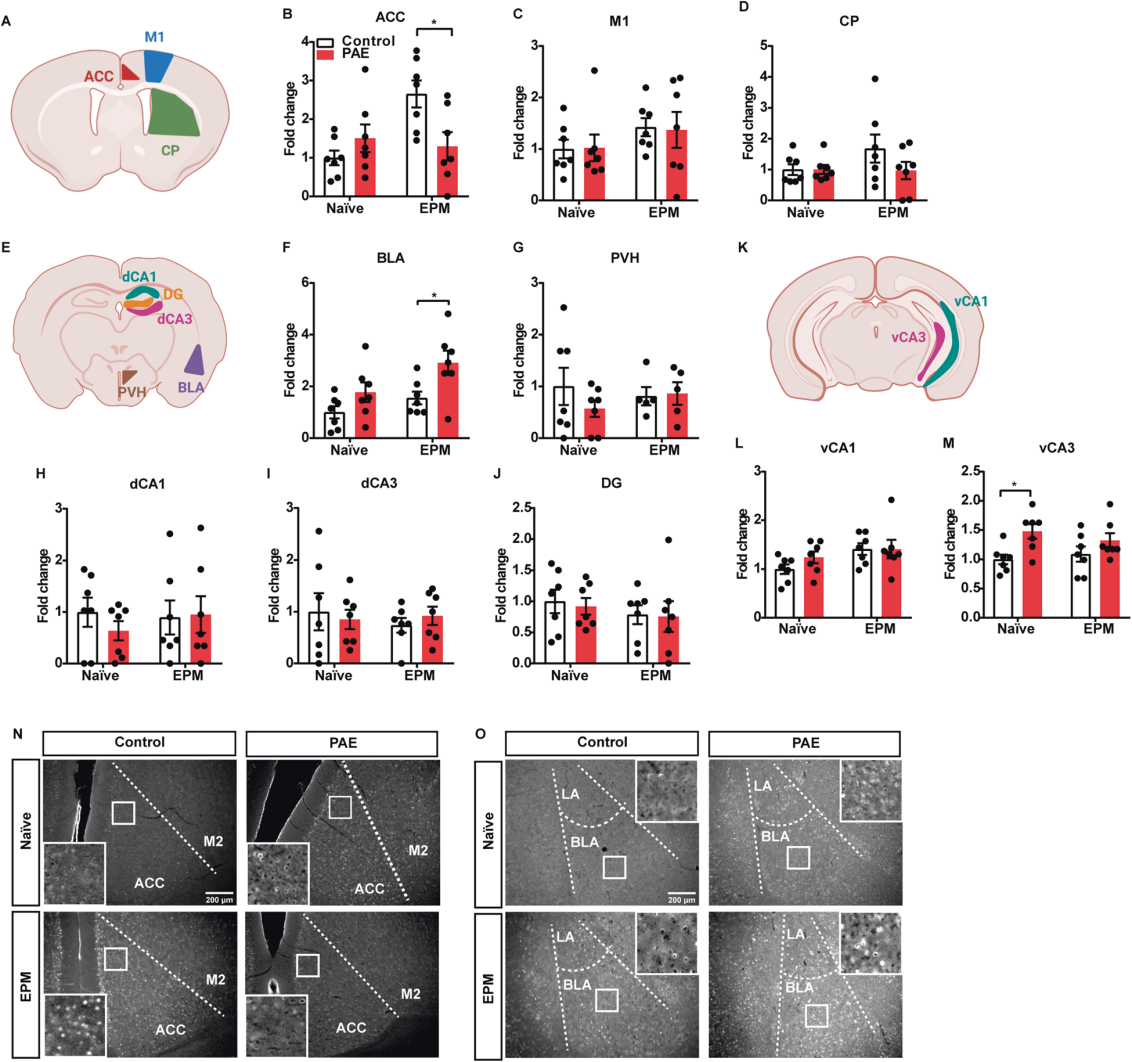
Neuronal activity in brain regions that are associated with anxiety behavior is altered in the PAE mice. **A**, **E**, **K** c-Fos was detected by immunohistochemistry, and the number of c-Fos^+^ cells was quantified in the brain regions highlighted in the schematics. **B** In the ACC, a significant interaction between the exposure and behavior test was observed, *F*(1,24) = 8.257, *p* = 0.0084 by two-way ANOVA. After the EPM test, the number of c-Fos^+^ cells was reduced in PAE (*adj. *p* = 0.0137 by simple main effects test). **C**, **D** No difference was found in the M1 and CP by two-way ANOVA. **F** There were significant differences in the number of c-Fos^+^ cells between different treatments and between naïve and post EPM tests in the BLA region. *F*(1,24) = 9.428, *p* = 0.0052 and *F*(1,24) = 5.914, *p* = 0.0229, respectively by two-way ANOVA. No interaction between these two factors was observed. There was an increase in c-Fos^+^ cells in PAE mice after the EPM test (*adj. *p* = 0.0497, Tukey’s post hoc test). **G**–**J** No significant changes in the PVH, dCA1, dCA3, or DG regions were observed. **L** No statistical significance in the vCA1 region was observed. **M** In the vCA3 region, there was a significant difference in the number of c-Fos^+^ cells between indicated exposure types. *F*(1,24) = 9.305, *p* = 0.0055 by two-way ANOVA. No interaction between the two measures was detected. Tukey’s post hoc test revealed a significant increase in c-Fos^+^ cells in the naïve PAE mice (*adj. *p* = 0.0367) compared to the naïve control mice. **B**–**D**, **F**–**J**, **L**, **M** Data are expressed as fold change as compared to naïve control. Graphs represent mean fold change ± SEM, and each dot represents an individual animal. *n* = 7 per group except for PVH after the EPM test (*n* = 5 per group). **N**, **O** Representative images of the c-Fos immunohistochemistry in the ACC and the BLA, respectively, with higher magnification images in inserts. Scale bars = 200 µm. ACC anterior cingulate cortex, M1 primary motor cortex, CP caudoputamen, BLA basolateral amygdala, PVH paraventricular hypothalamus, dCA1 dorsal CA1 of the hippocampus, dCA3 dorsal CA3 of the hippocampus, DG dentate gyrus, vCA1 ventral CA1 of the hippocampus, vCA3 ventral CA3 of the hippocampus.

Next, a comprehensive analysis of neuronal activity was performed by quantifying the expression of an immediate-early gene, c-Fos, that is known as a marker for neuronal activity in brain regions that are involved in anxiety and stress responses. First, c-Fos expression was quantified in different brain regions of the naïve control and PAE mice that were not subjected to any behavioral tests. Of all examined brain regions, there was a significant increase in the number of c-Fos^+^ cells only in the ventral CA3 region of the hippocampus (vCA3) of the PAE mice, relative to their controls (Fig. 2C). The vCA3 receives input from the amygdala, and activation of this circuitry increases anxiety-like behaviors [35, 36]. Thus, the slight increase of c-Fos, although not statistically significant, in the BLA between control and PAE with no behavior test (Fig. 2B) coincides with a corresponding significant increase in vCA3 (Fig. 2C) suggests potentially unregulated activation of BLA-vCA3 circuitry. This enhanced activity of BLA-vCA3 circuitry may have contributed to the activation HPA axis in the resting state and caused the release of high CORT in the PAE mice that were not put into the EPM test (Fig. 1I). Of note, no other brain regions showed changes in the c-Fos expression by PAE with no behavior test. Those results suggest that overall brain activities in the resting state were not significantly affected by PAE.

c-Fos expression was also examined 60 min post EPM test. While the number of c-Fos^+^ cells was significantly decreased in the ACC of the PAE mice (Fig. 2A), a significant increase in c-Fos^+^ cells was observed in their BLA (Fig. 2B). Studies have shown the functional connectivity between ACC and BLA [15, 16, 18, 37]. Therefore, our data suggest that anxiety-like behavior in the PAE mice (Fig. 1) is associated with changes in neuronal activity of BLA-projecting ACC neurons.

### Imbalance of excitatory and inhibitory activities in the ACC in the PAE

**Fig. 3:**
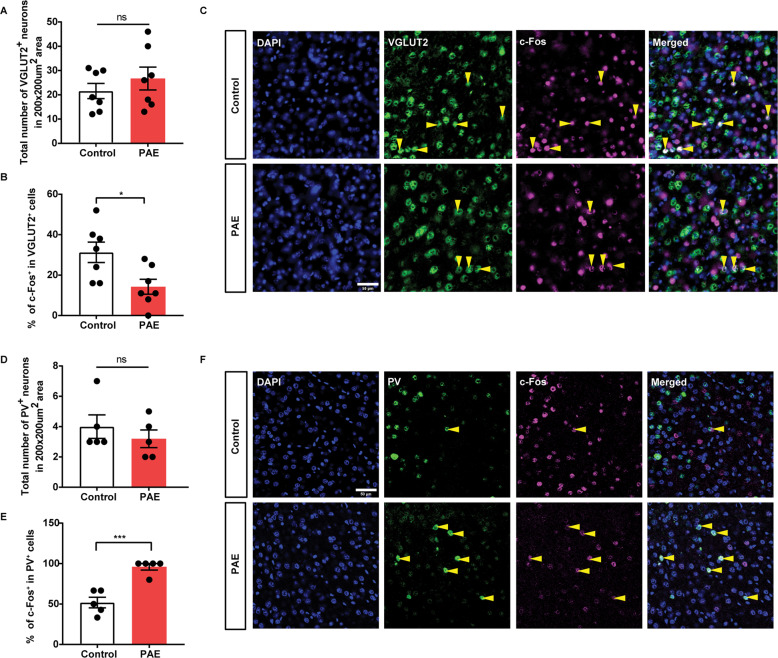
Imbalance of excitatory and inhibitory activities in the ACC in the PAE mice. Immunohistochemistry was performed to detect activation of excitatory (VGLUT2^+^) or inhibitory (PV^+^) neurons via c-Fos staining in the animals 60 min after the EPM test, and the number of cells with colocalized signals was quantified in the ACC. **A**, **B** There was no difference in the total number of VGLUT2^+^ cells between control and PAE mice. PAE mice showed a significantly lower number of VGLUT2^+^ neurons that are also c-Fos^+^ compared to control mice (*p = 0.0188). *n* = 7 mice each. **C** Representative images of immunohistochemical detection of VGLUT2 and c-Fos on the coronal brain slices from the PAE and the control mice post EPM test. Yellow arrowheads indicate cells that express both VGLUT2 and c-Fos. Scale bar = 50 µm. **D**, **E** No difference in the total number of PV^+^ cells between control and PAE mice was observed. The number of PV^+^ cells that co-express c-Fos was significantly reduced in the PAE (***p = 0.0004). *n* = 5 mice each. **F** Representative images of PV and c-Fos colocalized cells. Scale bar = 50 µm. **A**, **B**, **D**, **E** Graphs represent mean ± SEM, and each dot represents an individual animal. Unpaired two-tailed Student’s *t*-test. ns not significant.

We then examined the underlying mechanisms that could alter the activity of ACC neurons innervating BLA in the PAE mice. FASD preclinical models show changes in both excitatory and inhibitory neurons in terms of neuronal activity and gene expression. A maternal binge-drinking regimen, that is similar to ours, results in upregulated activities of parvalbumin (PV)^+^ interneurons, a subtype of inhibitory interneurons, in the ACC [38]. Moreover, neonatal alcohol exposure results in a decrease in the number of PV^+^ interneurons in the somatosensory cortex [39], and an overall reduction in GABAergic interneurons in the hippocampus in mice [40]. In excitatory neurons in another PAE model, mRNA expression of vesicular glutamate transporter 2 (VGLUT2) was increased but the protein level was decreased instead. In the same animals, vesicular glutamate transporter 1 (VGLUT1) expression level was not altered [41]. Given that a reduction in the number of VGLUT2 expressing neurons in the cortical area leads to an anxiolytic effect [42], the changes in the number of VGLUT2 expressing cells in the ACC may alter anxiety behavior in our PAE model. Based on the previous findings, the total number of VGLUT2^+^ projecting excitatory neurons or PV^+^ inhibitory interneurons was examined in the ACC 60 min after the EPM tests, but no difference was found between PAE and control (Fig. 3A, D). However, we found that the activities of the VGLUT2^+^ projecting excitatory neurons and PV^+^ inhibitory interneurons were altered (Fig. 3B, E). By double immunolabeling these neuronal subtypes with c-Fos in the ACC of mice subjected to the EPM tests, we found that the percentage of c-Fos^+^ cells was significantly decreased in the VGLUT2^+^ projecting excitatory neurons in the PAE (Fig. 3B, C). In contrast, the percentage of c-Fos^+^ cells was significantly increased in the PV^+^ inhibitory interneurons in the PAE (Fig. 3E, F). We also examined other subtypes of interneurons (calretinin, VIP, and SST) and found that there are no significant changes in the number of those neuronal subtypes (Supplementary Fig. 3). In addition, there was a very few c-Fos^+^ expression in those neurons (Supplementary Fig. 3), demonstrating that the ACC activity is reduced primarily due to the changes in the PV^+^ neurons.

### Chemogenetic activation of excitatory neurons in the ACC reduces anxiety in the PAE mice

**Fig. 4:**
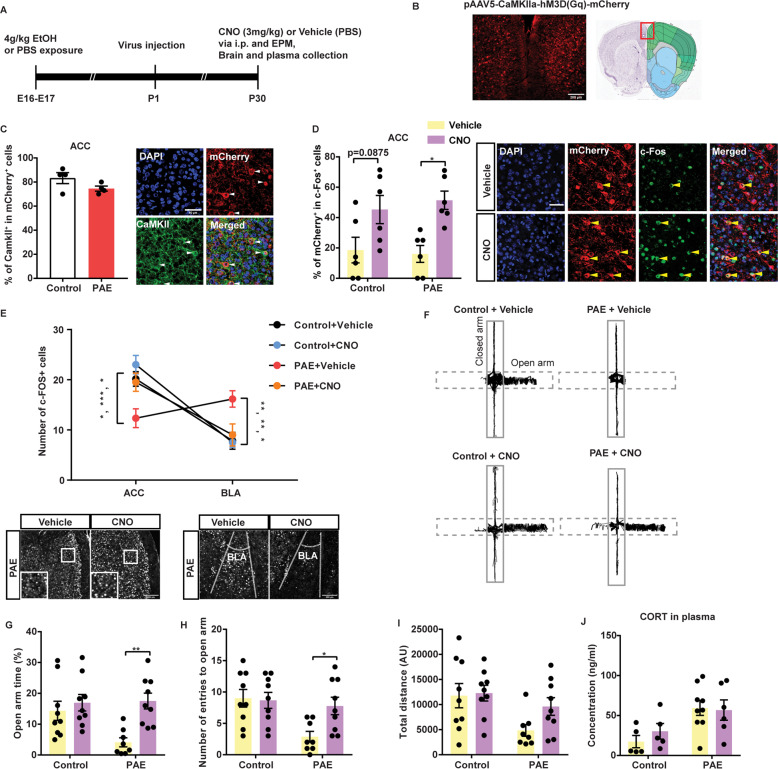
Chemogenetic activation of excitatory neurons in the ACC reduces anxiety in the PAE mice. **A** Experimental timeline. **B** Representative image of the ACC expressing mCherry 30 days after injection of CaMKIIa-hM3D(Gq)-mCherry AAV. The red box in the coronal section diagram indicates the corresponding brain area in the image. **C** The majority of cells expressing mCherry were also immunolabeled with a pan-CaMKII antibody, confirming the specificity of DREADD in the excitatory neurons in the ACC. *n* = 4 mice per group. There was no significant difference in CaMKII positivity ratio in mCherry^+^ cells between control and PAE (unpaired two-tailed Student’s *t*-test). Representative images are shown on the right. White arrowheads indicate mCherry and CaMKII co-expressing cells. **D** After CNO administration, the number of c-Fos^+^ cells was significantly increased among mCherry-positive cells in the ACC of DREADD animals. *F*(1,20) = 17.12, *p* = 0.0005 in administration type (CNO vs. vehicle), but no significance was observed in exposure type (control vs. PAE) by two-way ANOVA. The number of mCherry-positive cells that express c-Fos was significantly higher in the CNO-administered PAE mice compared to the vehicle-administered PAE mice (*adj. *p* = 0.0161) by Tukey’s post hoc test. Representative images of ACC on the right showing cells co-labeled with c-Fos and mCherry after vehicle (top) or CNO (bottom) administration (arrowheads). **E** The numbers of c-Fos^+^ cells in 200 µm^2^ were counted in the ACC and the BLA after indicated treatments. (3,40) = 12.54, *p* < 0.0001 by two-way ANOVA. ACC: Control+Vehicle vs. PAE + Vehicle *adj. *p* = 0.0123, Control + CNO vs. PAE + Vehicle ***adj. *p* = 0.0004. BLA: Control+Vehicle vs PAE + Vehicle **adj. *p* = 0.0071, Control+CNO vs. PAE + Vehicle **adj. *p* = 0.0048 by simple main effect tests. In the PAE mice, CNO-driven neuronal activation significantly increased the number of c-Fos^+^ cells in the ACC (*adj. *p* = 0.0251), while the corresponding number decreased in the BLA (*adj. *p* = 0.0251) compared to that of vehicle administration. Representative immunohistochemistry of c-Fos probed ACC (left) and BLA (right) of vehicle or CNO administered PAE mice are shown at the bottom. **F** Representative images of line tracks generated by MouBeat ImageJ plug-in from a recording of a mouse in the EPM test. Dashed and solid boxes outline open and closed arms, respectively. **G**–**J** DREADD reduced anxiety phenotype in PAE. Interactions were significant between administration type and exposure group in both duration (**G**) and number (**J**) of entries into the open arm. *F*(1,31) = 4.313, *p* = 0.0462 and *F*(1,31) = 4.191, *p* = 0.0492, respectively by two-way ANOVA. DREADD by CNO injection significantly increased time in the open arm (**adj. *p* = 0.0022 by simple main effect test) and the number of entries to the open arm (*adj. *p* = 0.0451) in PAE mice. However, DREADD did not affect the total distance traveled in the EPM test or CORT concentration in the plasma. Exposure type: *F*(1,31) = 6.97, *p* = 0.0129 and *F*(1,21) = 10.38, *p* = 0.0041 by two-way ANOVA, respectively. Treatment: not significant. **D**, **E** *n* = 6 per group. **G**–**I** Control + Vehicle *n* = 9, PAE + Vehicle *n* = 8, Control + CNO *n* = 9, PAE + CNO *n* = 9. **J** Control + Vehicle *n* = 5, PAE + Vehicle *n* = 9, Control + CNO *n* = 5, PAE + CNO *n* = 6. Graphs represent mean ± SEM, and each dot represents an individual animal. Scale bars = 50 (**C**, **D**), 200 µm (**B**, **E**).

To test whether the reduced activity of excitatory neurons in the ACC contributes to increased anxiety in the PAE mice, the Designer Receptors Exclusively Activated by Designer Drugs (DREADD) method was employed as outlined in Fig. 4A. To specifically express DREADD in excitatory neurons in the ACC, AAV5-CaMKIIa-hM3D(Gq)-mCherry virus was injected bilaterally to the ACC in control and PAE pups at P1. Expression of mCherry was confirmed in the ACC in virus-injected mice at P30 (Fig. 4B), and immunohistochemical detection of pan-CaMKII and mCherry verified the selective expression of DREADD in excitatory neurons (Fig. 4C).

At P30, hM3Dq virus-injected mice were subjected to the EPM test 30 min after administration of the Clozapine N-oxide CNO (3 mg/kg) or vehicle, and plasma samples and brain tissues were collected 60 min post EPM test. After CNO administration, a similar level of activation of mCherry^+^ neurons was confirmed by elevation of c-Fos expression in the ACC for both control and PAE mice (Fig. 4D). CNO administration also increased c-Fos^+^ cell density in the ACC of hM3Dq-injected PAE mice accompanied by a reduction in density of c-Fos^+^ cells in the BLA, compared to vehicle-administered hM3Dq-injected PAE mice (Fig. 4E).

In the PAE, DREADD-mediated chemogenetic activation of excitatory neurons in the ACC significantly increased time spent in the open arm (Fig. 4F, G) and the number of entries to the open arm (Fig. 4H). Although not statistically significant, the PAE mice administered with CNO showed a trend towards an increase in the total traveling distance (Fig. 4I). In the control mice, there was no significant difference between CNO- and vehicle-administered mice in any of these parameters, demonstrating that either CNO administration at the indicated concentration or viral injection did not change animal behavior in normal mice. To rule out a possible effect of CNO that can convert back to clozapine to become a potent antipsychotic binding to receptors in neurons to alter behavior [43], the EPM test was administered to CNO-injected PAE mice that did not receive DREADD viral injection (Supplementary Fig. 2). Compared to vehicle-administered animals, the performances of those animals are similar, therefore, the effects observed in Fig. 4G, H are independent of clozapine activity.

Notably, anxiolytic effects were mediated by activation of excitatory neurons in the ACC without changes in the CORT concentration in plasma from the PAE mice (Fig. 4J), indicating that ACC activation alone is not sufficient to reduce CORT release in the PAE mice. Altogether, these results demonstrate that chemogenetic activation of ACC mitigated anxiety phenotype in the PAE mice without changing the response of the HPA axis.

## Discussion

In this study, we show that PAE disrupts the response of the ACC against stressful events. Brain-wide analysis of c-Fos detection and the ancillary experiments revealed a strong functional association between ACC, BLA, and plasma CORT level as well as the anxiety behavior in the PAE (Figs. 1 and 2). In the PAE mice, DREADD-mediated activation of excitatory neurons in the ACC reduced both hyperexcitation of the BLA and anxiety behavior in the PAE mice (Fig. 4), underscoring the contribution of ACC in anxiety behavior in the PAE.

A similar reduction of activity in the prefrontal cortex (PFC) was observed in another study where the mice were exposed to ethanol for a shorter duration and lower concentration around the same developmental stage. In these mice, the number of PV^+^ inhibitory interneurons was increased at P70, and the excitatory-inhibitory balance was shifted towards inhibition in the medial PFC (mPFC) [38]. These mice also presented cognitive inflexibility and hyperactivity [38]. In this study, PV immunoreactivity in the PAE mice was the same as in control mice at P30. However, enhanced activity in PV^+^ interneurons and an overall reduction of excitation in the ACC were observed (Fig. 3). Although these mice did not demonstrate hyperactivity [23], they showed anxiety behavior (Fig. 1) and cognitive inflexibility (manuscript in preparation). In contrast to the observation made at P30, the brains from the PAE mice at P90 that were not placed in any behavior experiments showed a significant increase in the number of PV^+^ interneurons in the ACC compared to the control mice (Supplementary Fig. 4), suggesting age-dependent differences in the PAE phenotypes.

In this study, VGLUT2 was used as a marker for glutamatergic neurons. We found that the activities of VGLUT2^+^ neurons are significantly decreased in the PAE compared to the control after the EPM test (Fig. 3). As other glutamatergic neurons such as VGLUT1 expressing neurons are not formally tested in this study, we cannot rule out the changes in the activities of other types of glutamatergic neurons in the PAE.

The elevated plasma CORT in naïve PAE mice suggests chronic exposure to a high level of CORT compared to control mice (Fig. 1I). Of note, a mouse model of chronic exposure to restraining stress showed a reduction in the activity of the PFC with elevated number and activation of PV expressing interneurons [44, 45]. One of those studies also showed that chemogenetic activation of PV^+^ interneurons resulted in an overall reduction of PFC activity and elicited anxiety-related behavior [44]. Chronic exposure to CORT, either induced by daily restraining or direct injection of CORT into rats for a prolonged period, also decreased the complexity of apical dendrites of pyramidal neurons in the PFC and synaptic transmission in other brain regions such as the dorsal raphe nucleus in the midbrain [46–50]. Based on those findings, a hypothetical mechanism underlying ACC-mediated anxiety in the PAE is that increase in glucocorticoid level causes a reduction in activity and morphological complexity of the excitatory neurons in the ACC. Given the glucocorticoid receptors are expressed in both excitatory and inhibitory neurons in the ACC [51, 52], those changes may be caused directly by binding of CORT on the receptors in the excitatory neurons or/and indirectly through hyperactivation of the PV expressing interneurons by chronic CORT exposure (Figs. 1I and 2).

As downstream circuitry of ACC-BLA, it is known that excitatory neurons in BLA directly innervate the central amygdala (CeA). A few papers reported that glucocorticoids can directly affect CeA to increase anxiety-like behavior in the EPM tests [53–55]. Despite the high CORT level being sustained even after DREADD activation of ACC, the anxiety-like behavior was diminished (Fig. 4G, J). This result suggests that the projecting neurons from BLA to CeA dominate the function over the neurons that express glucocorticoid receptors in CeA. Another targeting brain area of the ACC is the paraventricular hypothalamic nucleus (PVH), where corticotropin-releasing factor (CRF) is released upon restraining stress to stimulate CORT synthesis in the adrenal cortex [56]. Ablation of the dorsal mPFC, where ACC is included, stimulated the PVH to produce more CRF than normal conditions under restraint stress, suggesting negative regulation of the ACC on the CORT synthesis in response to stress exposure. Therefore, we anticipated that chemogenetically introduced restoration of the ACC activity would decrease the plasma CORT level; however, it did not (Fig. 4). This discrepancy may be due to the chemogenetic activation being too short to induce detectable changes in the CORT level. However, this result also points out that, regardless of CORT level, the activation of the downstream target of ACC can improve the anxiety phenotype (Fig. 4). Altogether, our results indicate a potential of transcranial direct current stimulation used for attention deficits in FASD patients [57], as a treatment option targeting ACC for emotional problems in these patients.

## Supplementary information

Supplementary figure legends

Supplemental Fig 1

Supplemental Fig 2

Supplemental Fig 3

Supplemental Fig 4
