# Supplementary material for: Activation of the anterior cingulate cortex ameliorates anxiety in a preclinical model of fetal alcohol spectrum disorders
Source: Transl Psychiatry. 2022 Jan 20;12:24. doi: 10.1038/s41398-022-01789-1 (PMC8776849; doi:10.1038/s41398-022-01789-1)
Supplement: Supplementary file 1 — Supplementary figure legends [file 41398_2022_1789_MOESM1_ESM.docx]

**Supplementary Figure 1. Evaluation of sexual dimorphism in behavioral tests and plasma CORT analysis.**

To determine if sexual dimorphism exists in the phenotypes studied here, the behavioral and CORT data from animals used in Fig.1 were divided by sex for each group (control and PAE), and a two-tailed Student’s t-test was performed to compare between data derived from males and females. The analysis showed that there is no difference between the sexes in self-grooming behavior (A), EPM test (B), or fold change in CORT concentrations in the plasma from naïve and post EPM test mice (C). (A) n=7 per group per sex. (B) male n=4 and female n=5 per group. (C) Naïve: control male = 6, control female=6, PAE male=4, PAE female=5. EPM: control male =5, control female n=5, PAE male=4, PAE female n=5. ns = not significant. Graphs represent mean +/- SEM, and each dot represents an individual animal.

**Supplementary Figure 2. CNO administration alone does not affect mouse behavior in the EPM test.**

In the EPM test, CNO administered PAE mice without DREADD virus injection showed no difference in the open arm time (A), number of entries to open arm (B), and total traveling distance (C) compared to vehicle administered DREADD injected PAE mice shown in Figure 4. CNO only n=5, DREADD+Vehicle n=8. ns = not significant by two-tailed Student’s t-test. Graphs represent mean +/- SEM, and each dot represents an individual animal.

**Supplementary Fig 3. PAE does not change the numbers or activities of the interneuronal subtypes at P30.**

Brains collected from P30 mice 60 minutes after completion of the EPM tests were immunolabeled with calretinin, vasoactive intestinal peptide (VIP), or somatostatin (SST) antibodies, and immunoreactive cells were counted in the ACC. (A, D and G) There are no significant changes in the number of immunoreactive cells with either of those three markers between control and PAE mice. (B, E, and H) There are very few colocalizations between the interneuronal markers and c-Fos without any statistically significant differences between control and PAE mice. (C, F, and I) Representative images of calretinin, VIP, and SST double-stained with c-Fos. Scale bar=20µm. Two-tailed Student’s t-test. Control n=6, PAE n=6. Graph represents mean +/- SEM, and each dot represents an individual animal.

**Supplementary Fig 4. The number of PV^+^ interneurons was increased in the ACC in PAE mice at P90.**

Brains from naïve control and PAE mice (animals that were not placed to behavior tests) at P90 were immunolabeled with PV antibody, and the number of PV^+^ cells was quantified in the ACC. (A) The number of PV^+^ interneurons is significantly increased in the PAE mice compared to the control (p=0.0002). Two-tailed Student’s t-test. Control n=6, PAE n=6. Graph represents mean +/- SEM, and each dot represents an individual animal.
