# Supplementary figures and images for: Activation of the anterior cingulate cortex ameliorates anxiety in a preclinical model of fetal alcohol spectrum disorders
Source: Transl Psychiatry. 2022 Jan 20;12:24. doi: 10.1038/s41398-022-01789-1 (PMC8776849; doi:10.1038/s41398-022-01789-1)

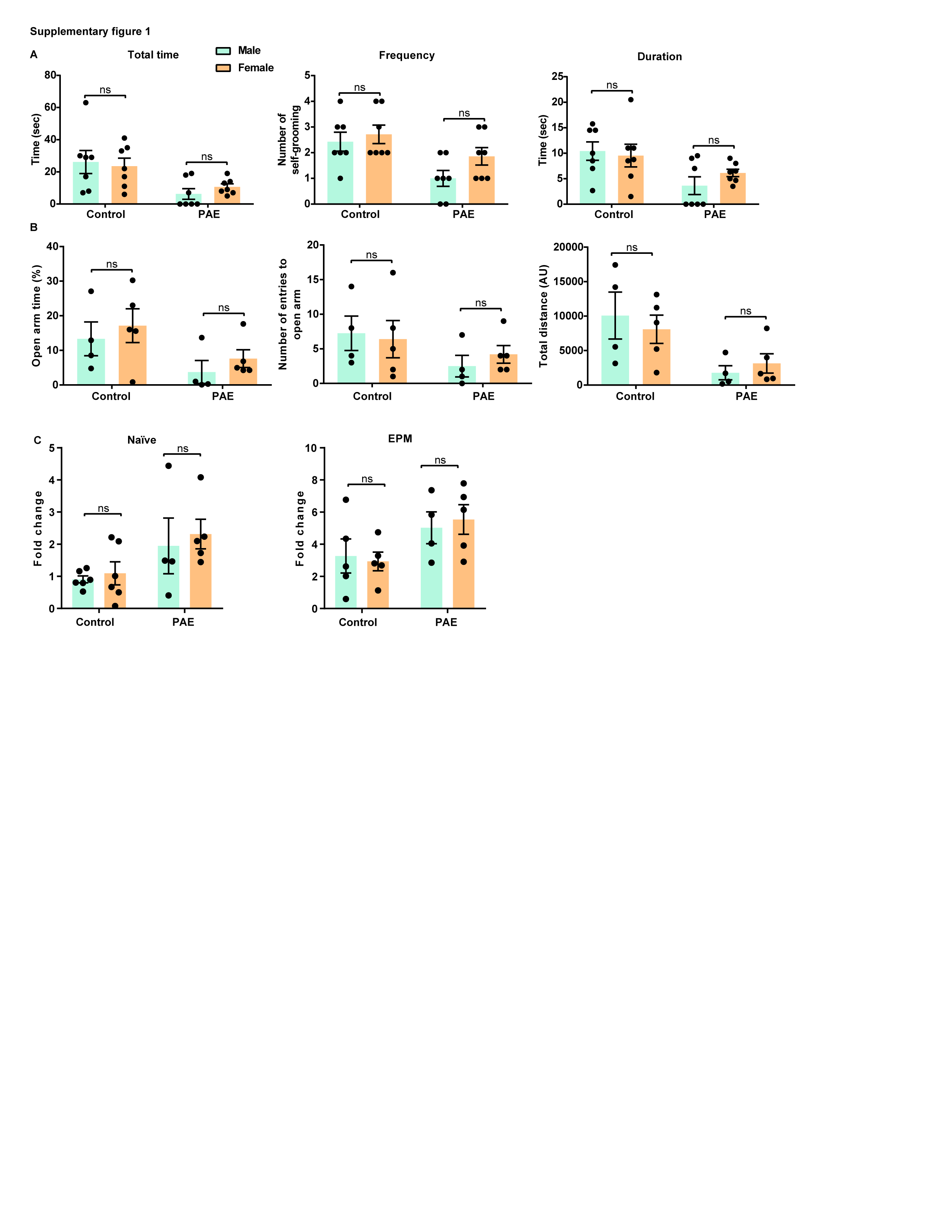

Supplement: Supplementary file 2 — Supplemental Fig 1 [file 41398_2022_1789_MOESM2_ESM.tif]

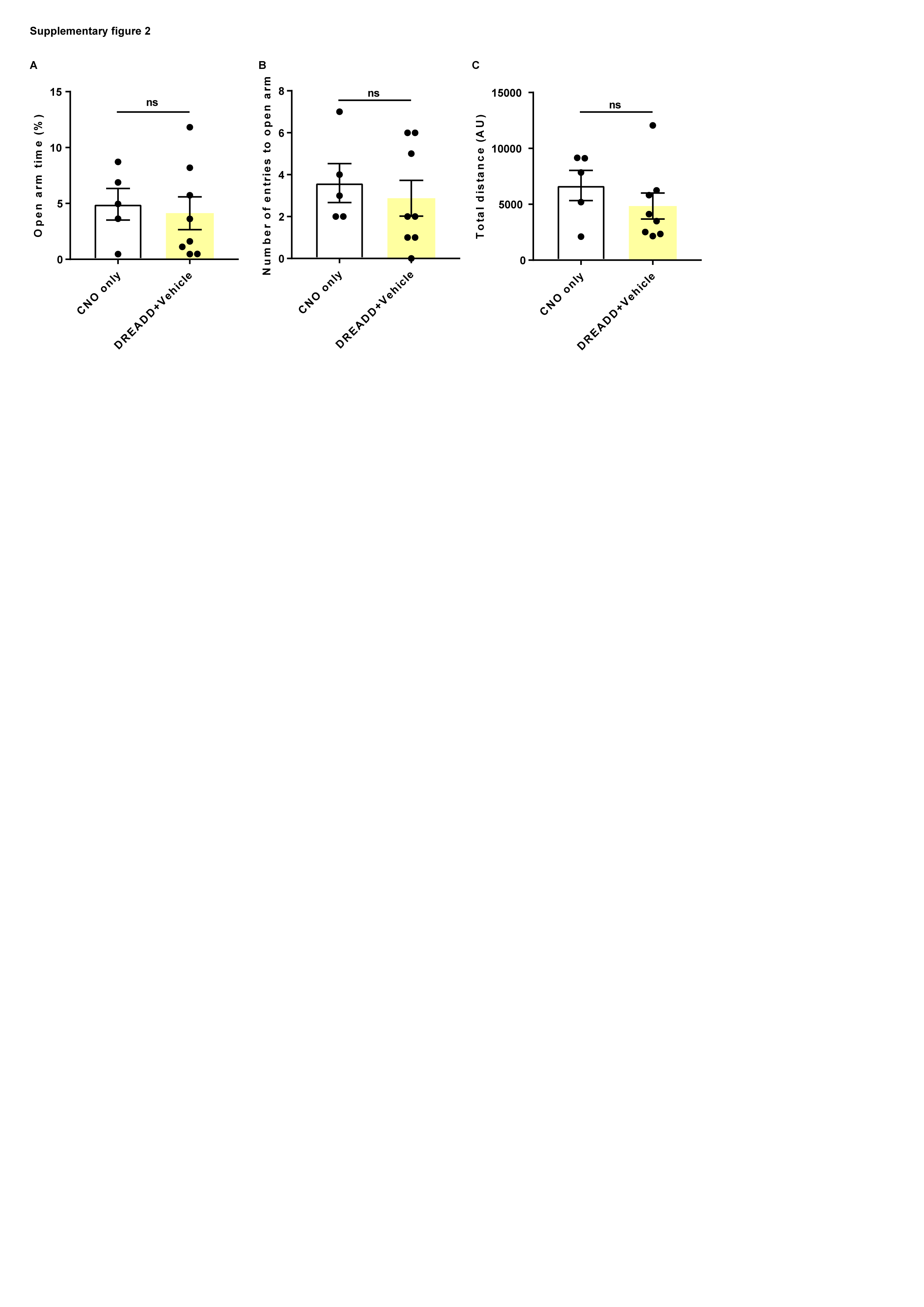

Supplement: Supplementary file 3 — Supplemental Fig 2 [file 41398_2022_1789_MOESM3_ESM.tif]

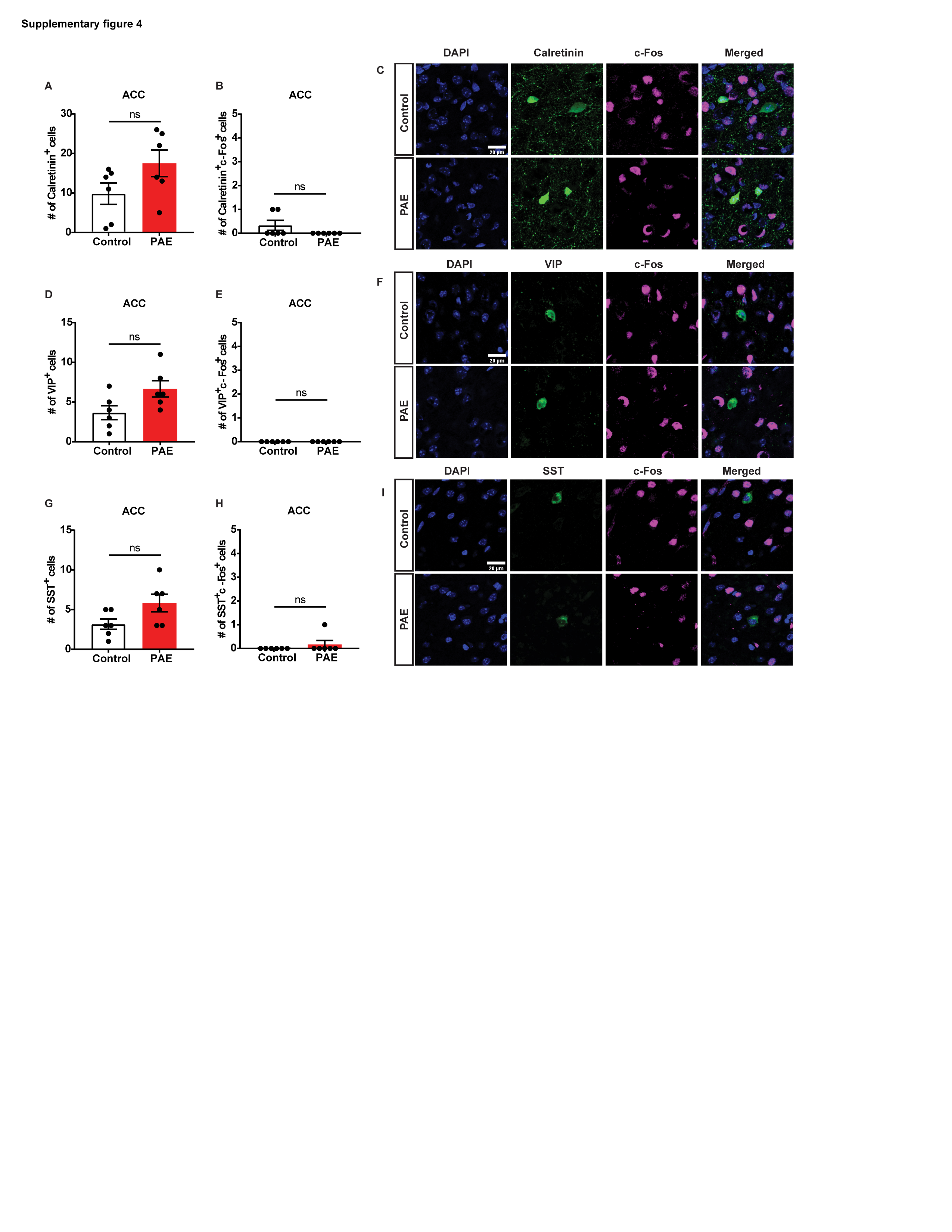

Supplement: Supplementary file 4 — Supplemental Fig 3 [file 41398_2022_1789_MOESM4_ESM.tif]

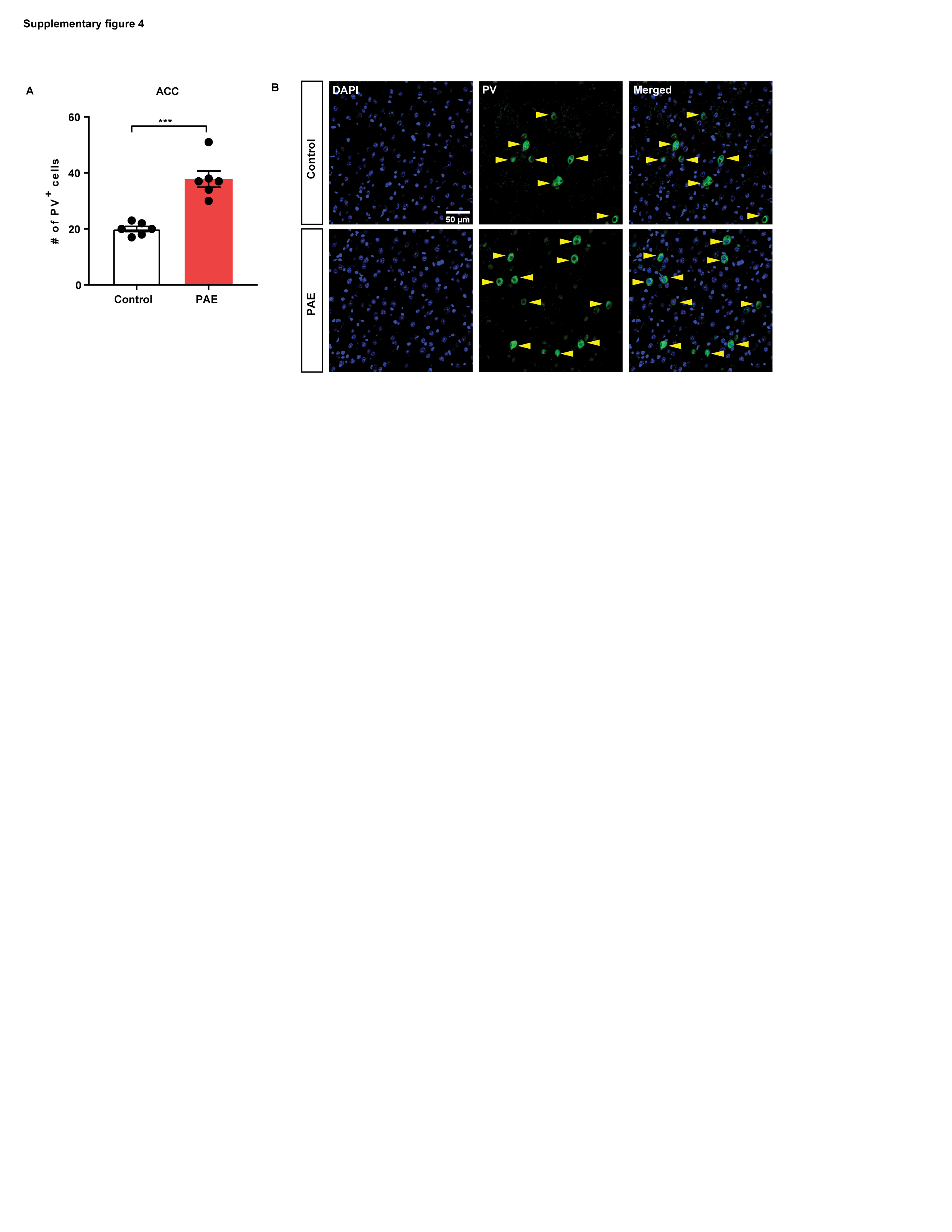

Supplement: Supplementary file 5 — Supplemental Fig 4 [file 41398_2022_1789_MOESM5_ESM.tif]
